# Bony prominence and variations of the transverse sinus groove: novel anatomical findings in relation to sinus pathologies

**DOI:** 10.1007/s10143-024-03072-x

**Published:** 2024-11-26

**Authors:** Juan J. Cardona, Joe Iwanaga, Arada Chaiyamoon, Arthur Wang, Christopher M. Nickele, Matthew R. Amans, Daniel M. Heiferman, Kendrick D. Johnson, Marios Loukas, Aaron S. Dumont, R. Shane Tubbs

**Affiliations:** 1 Department of Neurosurgery, Tulane Center for Clinical Neurosciences, Tulane University School of Medicine, 131 S. Robertson St. Suite 1300, New Orleans, LA 70112 USA; 2 Department of Structural & Cellular Biology, Tulane University School of Medicine, New Orleans, LA USA; 3 Department of Neurology, Tulane University School of Medicine, New Orleans, LA USA; 4 Department of Neurosurgery and Ochsner Neuroscience Institute, Ochsner Health System, New Orleans, LA USA; 5 Department of Anatomy, Division of Gross and Clinical Anatomy, Kurume University School of Medicine, Kurume, Japan; 6 Department of Anatomy, Faculty of Medicine, Khon Kaen University, Khon Kaen, Thailand; 7 Department of Neurosurgery, University of Tennessee Health Sciences Center, Memphis, TN USA; 8 Department of Radiology and Biomedical Imaging, University of California San Francisco, San Francisco, CA USA; 9 Department of Neurological Surgery, University of California San Francisco, San Francisco, CA USA; 10 Department of Neurosurgery, Endeavor Health, Naperville, IL USA; 11 Department of Anatomical Sciences, St. George’s University, St. George’s, Grenada; 12 Department of Surgery, Tulane University School of Medicine, New Orleans, LA USA; 13 University of Queensland, Brisbane, QLD Australia

**Keywords:** Transverse sinus, Dural venous sinus, Thrombosis, Stenosis, Intracranial hypertension, Anatomy, Cadaver

## Abstract

The transverse sinus (TS) is often involved with pathology for structural reasons. The aim of this study was to improve understanding of the anatomy along the groove of the TS and sigmoid sinus (SS), to discuss the relationship between the bony features and pathologies affecting the TS. Seventy dry skulls (140 sides) were used for detailed observation of the TS and SS grooves using gross investigation coupled with transillumination. Bony features such as the mastoid foramen (MF), occipital foramen (OF), granular foveolae (GF), and absence of the TS groove were evaluated, and a classification based on numbers of findings was proposed. The most common internal groove finding was MF (79.3%), followed by absent TS groove (32.9%), and OF (14.3%). MF was statistically more frequent on the left side (91.4%) than the right (67.1%) (*p* = 0.036); OF was statistically more frequent on the right side (24.3%) than the left (4.3%) (*p* = 0.008). Absent TS groove was more prevalent on the left side (54.3%) than the right (11.4%) (*p* = 0.000). A bony prominence (BP) was identified in 15.7% (11 skulls) and there were significant differences from the controls. The type III class (three internal groove findings) was the most prevalent, followed by type II (two findings). The complex and integral role of the bony features described here, and their particularities in normal patients or in those with DVS pathologies, is not well understood.

## Introduction

The transverse sinuses (TS) are parts of the dural venous sinuses (DVS) in the posterior cranial fossa. As important elements of the cerebrospinal venous system they help to drain blood, assisting the cranial component of venous outflow via the internal jugular vein, particularly in the supine position; in the erect position, the caudal component predominates via the vertebral venous plexus [1, 2]. Interestingly, Tucci et al. [3] recently reported a novel additional pathway for direct DVS drainage from the intracranium though the basilar venous plexus via the diploic space of the clivus. The TSs also receive tributaries from the superior petrosal sinuses, the inferior cerebral and cerebellar veins, and some diploic and occipital emissary veins. They originate at the confluence of sinuses near the internal occipital protuberance and course laterally along the occipital bone and the tentorium cerebelli, terminating in the sigmoid sinuses (SS), which curve inferomedially to reach the jugular foramen [1]. 

An important and frequently under-diagnosed pathology is cerebral vein and dural venous sinus thrombosis (CVST). Maali et al. [4] compared several features of CVST across continents, including common locations of thrombosis. The TS proved to be the most common CVST location globally, excluding Asia, where the most common CVST location was the superior sagittal sinus = 43% (followed by the TS with 29%), and North America where the TS = 32% and the superior sagittal sinus also = 32%. However, it is important to emphasize that the involvement of the TS is always greater when both sides are considered. Some studies have reported that the left TS is more often affected than the right, but the reason for this predilection has yet to be explored. Similarly, the greater prevalence of thrombosis in the TS than in other venous sinuses has not been explained [4–6]. Other pathologies involving the TS that have a multifactorial etiology and are associated with alterations in intracranial venous flow include venous sinus stenosis (VSS) resulting in pulsatile tinnitus and/or idiopathic intracranial hypertension (IIH) [7, 8]. We hypothesized that the morphology of the TS and the sigmoid sinus (SS) is associated with the reported pathologies. Therefore, the aim of this study was to clarify the anatomy of the TS and SS along the groove, considering important variables in the bone underlying the soft tissue, in order to discuss the relationship between the bony features and the potential development of various conditions affecting the DVSs such as CVST, VSS, and IIH.

## Materials and methods

### Morphometrical study using dry skulls

Seventy skulls (140 sides) derived from the osteological collection of Tulane University School of Medicine were used for detailed observations of the groove for the TS and SS by gross investigation coupled with transillumination in order to evaluate internal osseous differences.

1. Features and internal groove findings

**Fig. 1:**
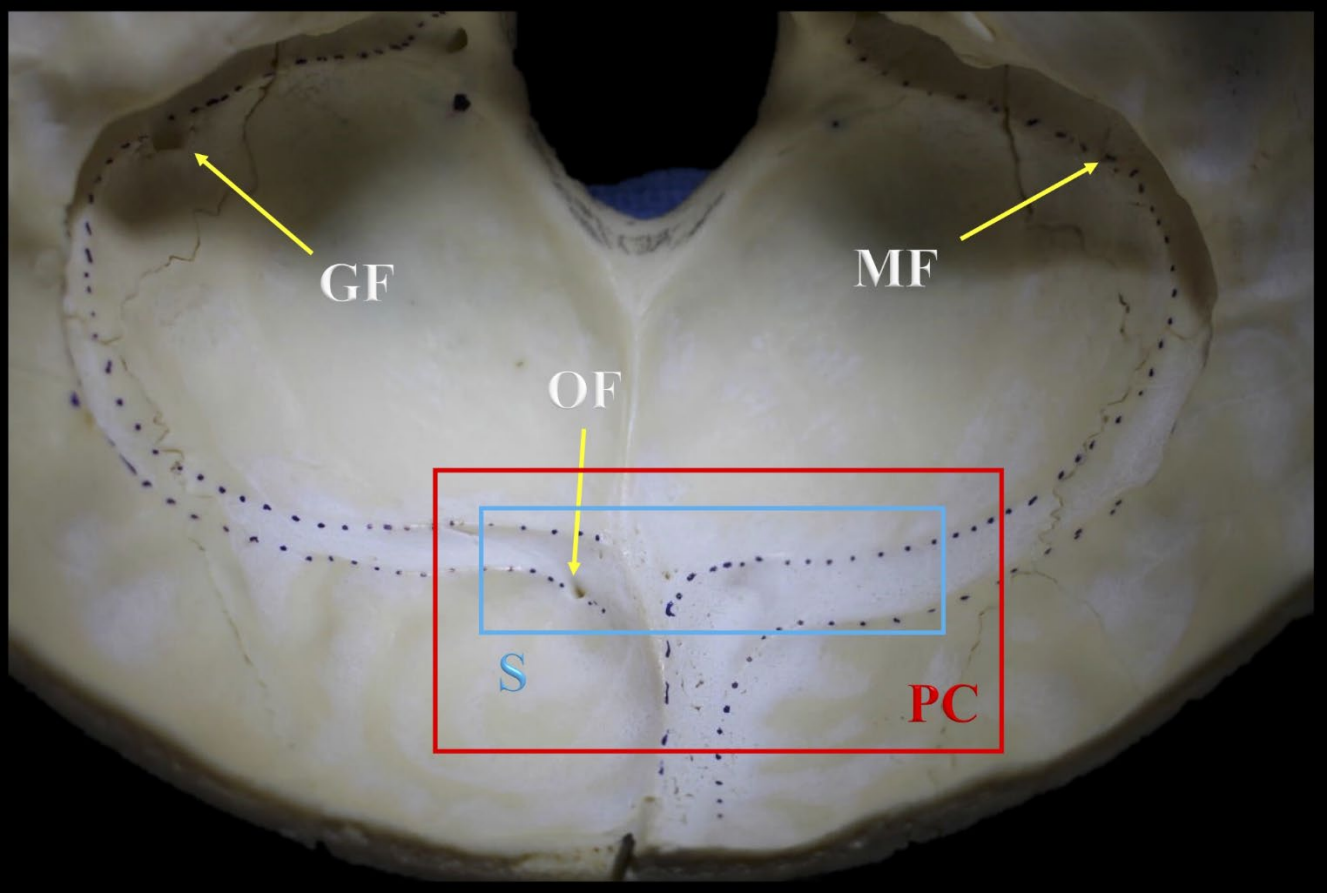
Example of a type III class and the bony parameters evaluated in the current study, precluding absent TS groove specimens. Note the symmetry (S) between the TS grooves seen almost at the same horizontal level (blue box) and an example of a right dominant pattern of communication (PC) at the confluence of the sinuses (red box). MF: Mastoid foramen; OF: Occipital foramen; GF: Granular foveolae

**Fig. 2:**
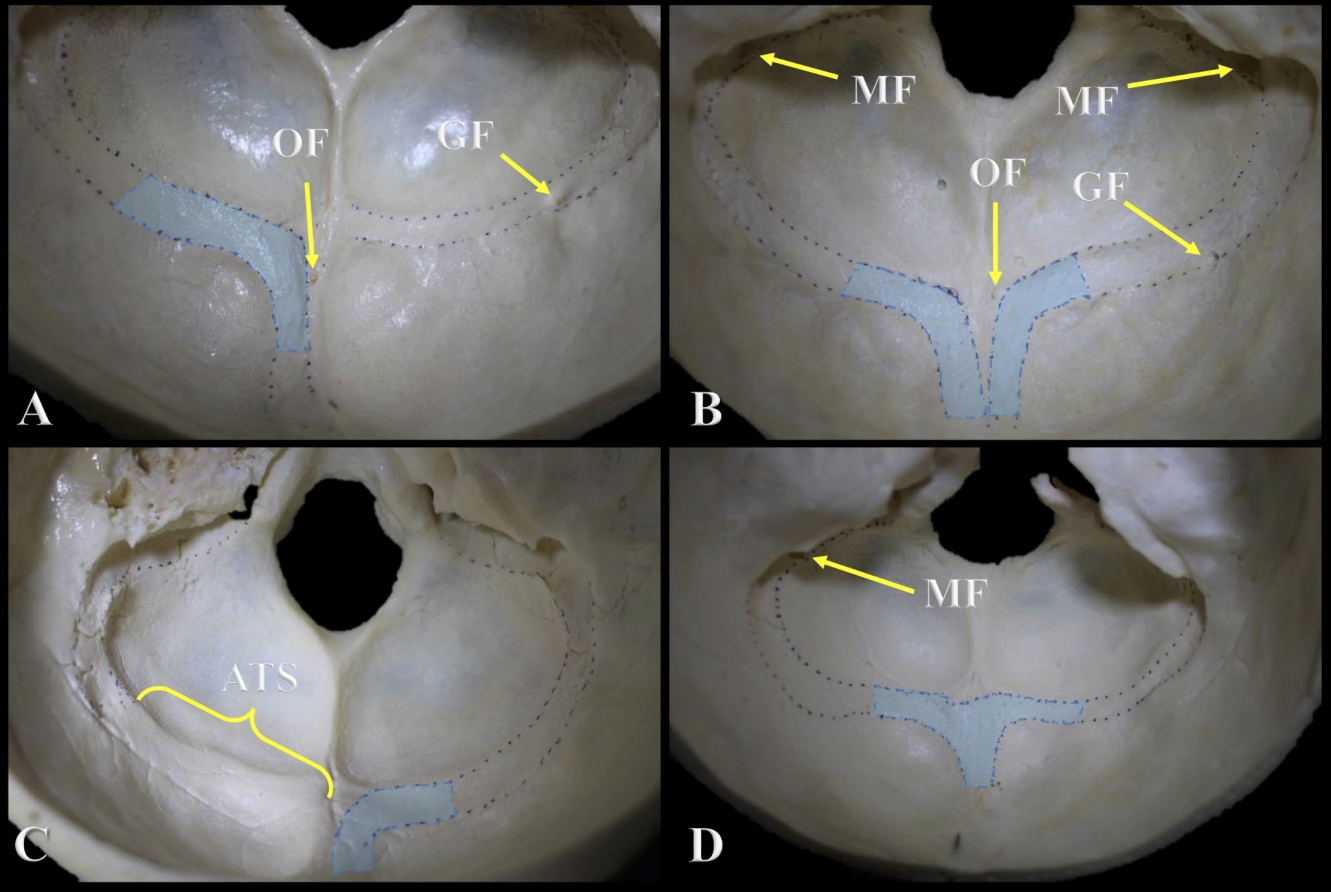
Pattern of communication at the confluence of the sinuses and examples of the classification based on number of internal groove findings. **A**: Left dominant pattern with two findings (Type II). **B**: Bifurcation pattern with four findings (Type IV). **C**: Right dominant pattern with one finding (Type I). **D**: Confluence pattern with one finding (Type I). OF: Occipital foramen; MF: Mastoid foramen; GF: Granular Foveolae; ATS: Absent Transverse Sinus Groove

The TS grooves were documented as symmetrical if they were at the same horizontal level, or as asymmetrical if they were not. The patterns of communication of the DVS grooves at the confluence of the sinuses were recorded and four groups were distinguished: right dominant, left dominant, confluence, and bifurcation. Bony features frequently found within 5 mm of the groove for the TS and SS including mastoid foramen (MF), occipital foramen (OF), granular foveolae (GF), and absence of TS groove were documented. The internal grooves were classified into five types (0, I, II, III, and IV) on the basis of the number of findings: 0: no findings; I: one finding; II: two findings; III: three findings; IV: more than three findings (Figs. 1 and 2).

2. Bony prominence located at the TS groove

**Fig. 3:**
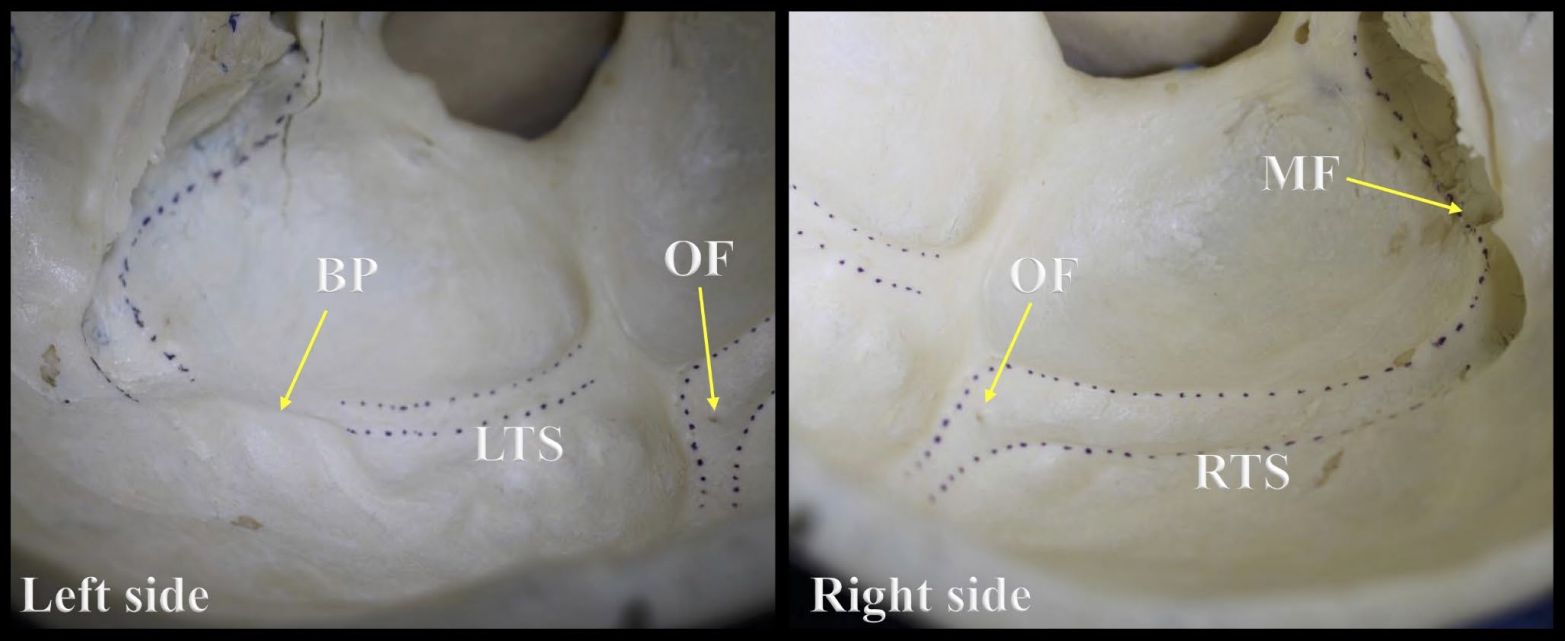
Example of a skull with a bony prominence on the left side. Note the difference between the left (LTS) and right (RTS) transverse sinus grooves. BP: Bony prominence; OF: Occipital foramen; MF: Mastoid foramen

**Fig. 4:**
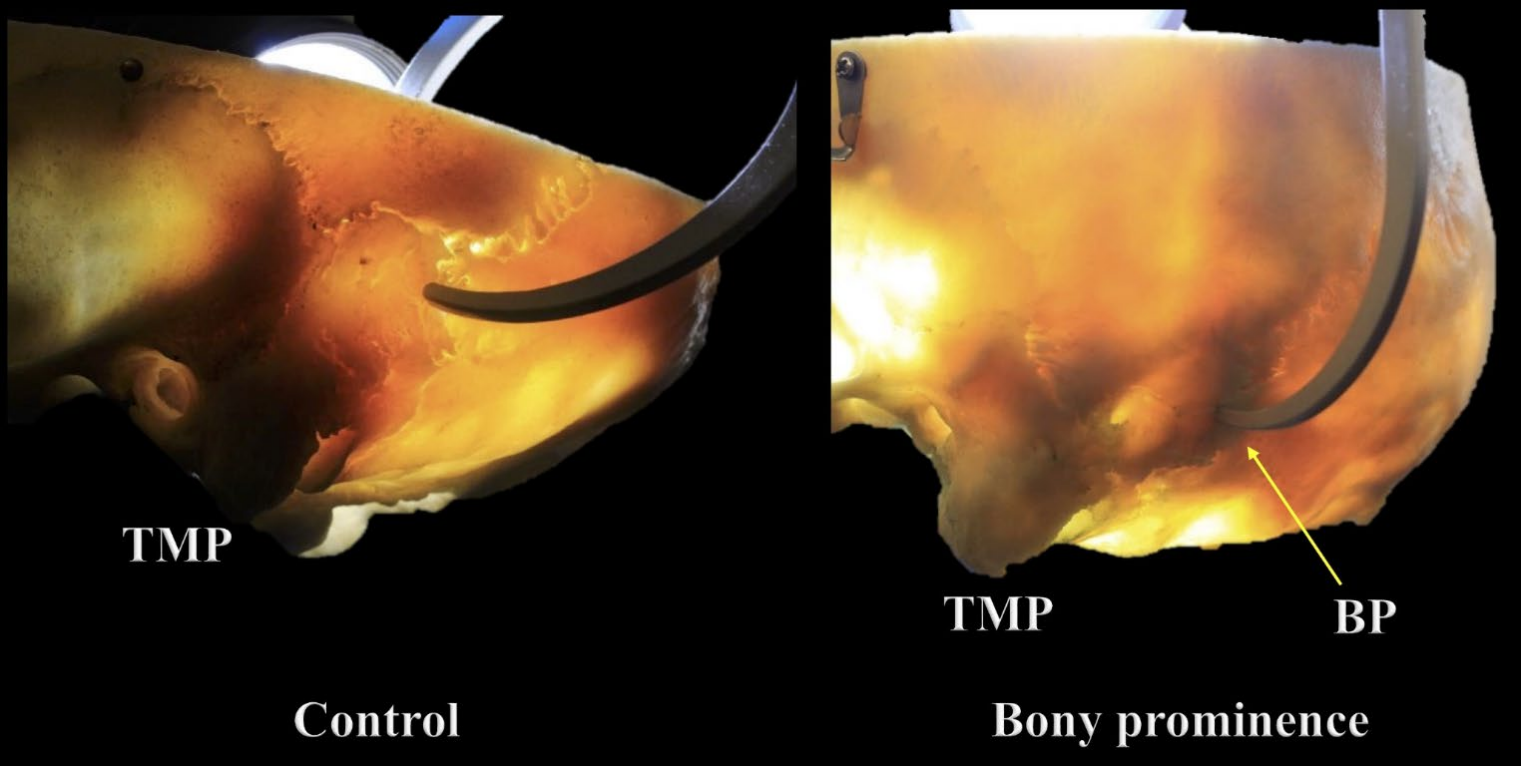
Internal bone difference between the bony prominence (BP) and control groups seen using transillumination. TMP: Tip of the mastoid process

During the gross examination and transillumination of the skulls with no TS groove, a novel finding was identified in some skulls and defined as a bony prominence (BP) (Figs. 3 and 4). All the specimens with a BP were collected, and the exact location and the thickness of the BP were documented. Selected specimens among the 70 skulls without BP and having a visible groove for the TS were used as controls to compare their thicknesses.

### Radiological study

Computed tomography (CT) scans were obtained for one control and one specimen, which had the thickest BP and significant internal osseous differences revealed by transillumination.

No sociodemographic data or clinical histories of the specimens were available. Microcalipers (Mitutoyo, Kawasaki, Japan) were used for triplicate measurements by two authors. SPSS software for Windows was used for statistical analyses of left and right side differences, with statistical significance set at *p* < 0.05. The authors state that every effort was made to follow all local and international ethical guidelines and laws that pertain to the use of human cadaveric donors in anatomical research [9]. 

## Results

### Parameters and internal groove findings

The most common pattern of communication at the confluence of the sinuses was right dominant (*n* = 56; 80.0%), followed by bifurcation (*n* = 4; 5.7%). The TS grooves were mostly symmetrical (*n* = 45; 63.6%), and when asymmetry was identified (*n* = 25; 36.4%) it was related in 14.3% (*n* = 10) of the specimens to absence of the TS groove, either in its proximal-middle segments or completely. The most common internal groove finding was MF (*n* = 111, 79.3%), which was always seen at the SS groove, followed by absent TS groove (*n* = 46, 32.9%), which mainly affected the middle-distal segments (*n* = 26; 18.6%), and OF (*n* = 20; 14.3%) located at the TS groove. Statistical analyses of side differences revealed a significant predominance MF on the left side (*n* = 64; 91.4%) over the right (*n* = 47; 67.1%) (*p* = 0.036). Also, OF, was significantly more common on the right side (*n* = 17; 24.3%) than the left (*n* = 3; 4.29%) (*p* = 0.008). TS groove was more often absent on the left side (*n* = 48; 54.3%) than the right (*n* = 8; 11.4%) (*p* = 0.000).

### Bony prominence located at the TS groove

**Fig. 5:**
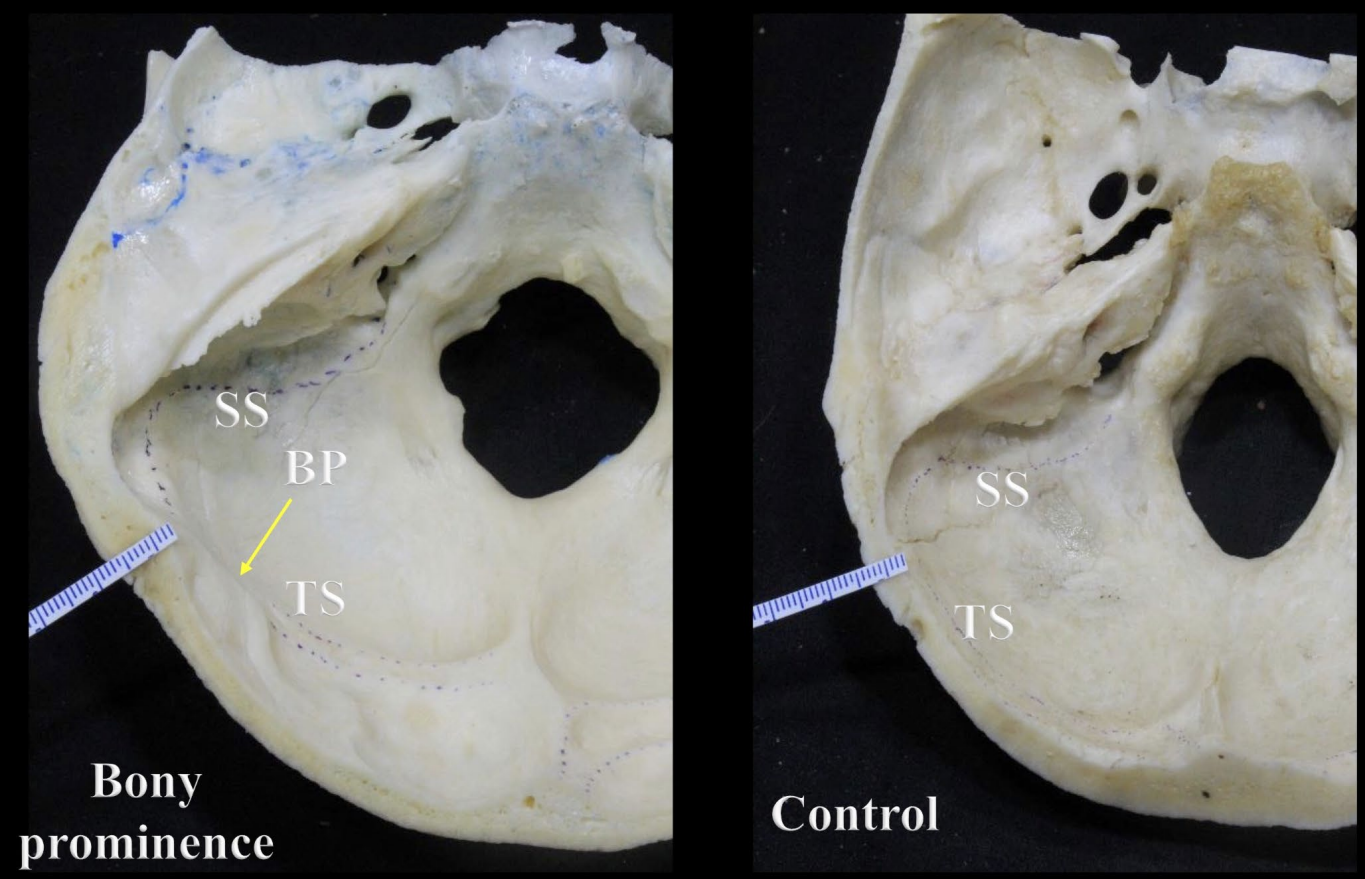
Transverse section through the skull showing the difference in thickness between the bony prominence (BP) and control groups. Note the sigmoid sinus (SS) and transverse sinus (TS) grooves

**Fig. 6:**
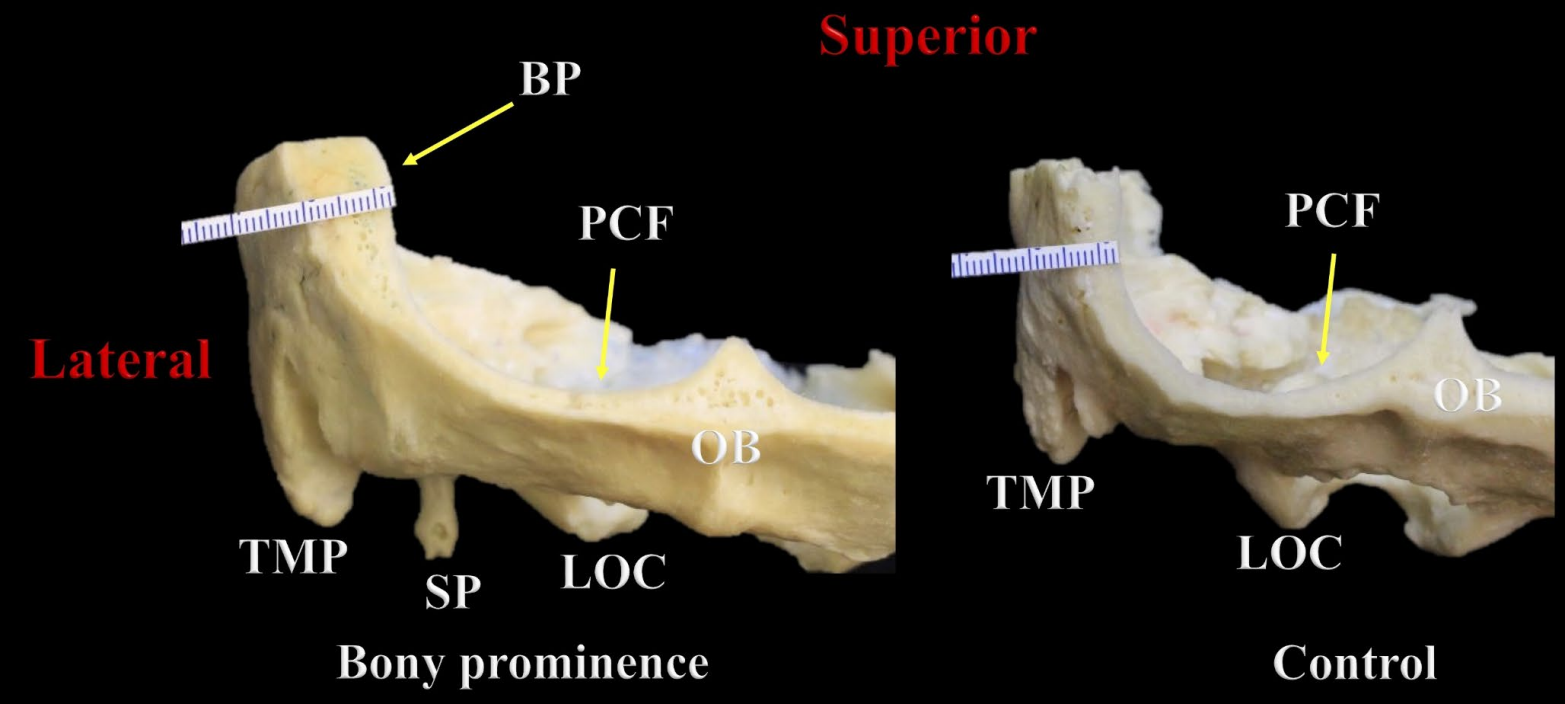
Coronal section through the skull at the posterior cranial fossa (PCF) showing the difference in thickness between the bony prominence (BP) and control groups. TMP: Tip of the mastoid process; SP: Styloid process; LOC: Left occipital condyle; OB: Occipital bone

**Fig. 7:**
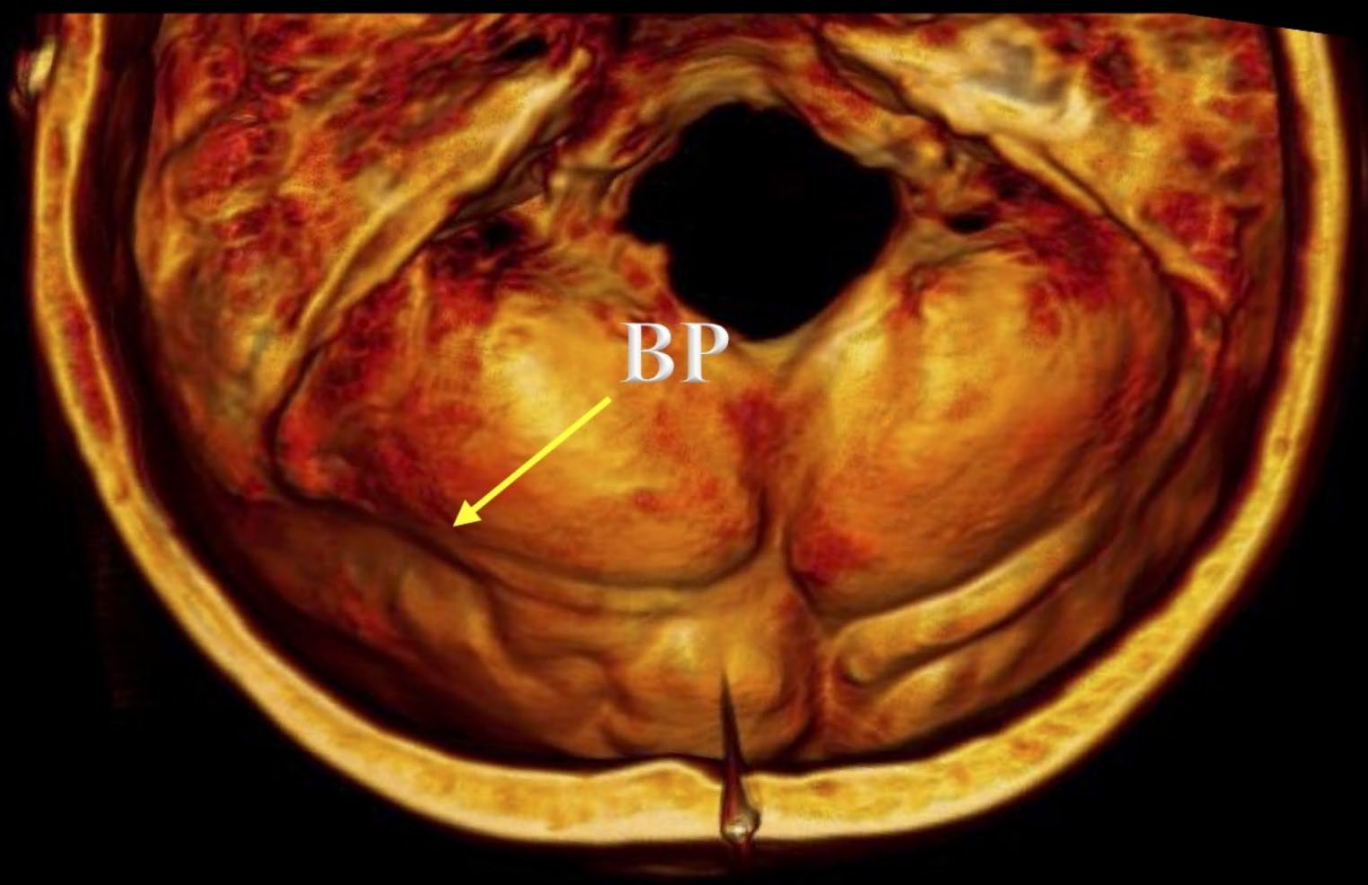
Difference in thickness between the bony prominence (BP) and control groups seen using CT scans

A BP was identified in 15.7% or 11 skulls. This finding mainly involved the middle-distal segments of the TS groove (*n* = 7; 63.6%), and it predominated significantly on the left side in skulls with a right dominant pattern (*n* = 10; 90.9%); there was one case of a BP located on the right side in a skull with a left dominant pattern (*p* = 0.000). The mean thicknesses of the BP and control groups were 6.88 ± 1.21 mm (range 4.4–9.4 mm) and 6.21 ± 0.49 mm (range 5.2–7.1 mm), respectively (Figs. 5 and 6). In the BP group, the left side (mean 7.74 ± 0.68 mm; range 7.0-9.4) was significantly thicker than the right (mean 6.01 ± 0.99 mm; range 4.4–7.6) (*p* = 0.000), presenting a mean left-right thickness difference of 1.73 ± 0.68 mm (range 0.6–2.6). Likewise, the mean difference in thickness between the BP and control groups was significantly greater on the left side (mean 1.68 ± 0.97 mm; range 0.2-4.0) than the right (mean − 0.34 ± 1.12 mm; range − 2.2/1.6) (*p* = 0.000). The bony differences seen in the BP area using transillumination were also revealed using CT (Fig. 7).

### Classification based on the number of internal groove findings

The most prevalent class was type III with three findings, followed by type II with two findings. When one finding (type I) was identified on the skulls, it was frequently related to absence of TS groove (Table 1).

**Table 1:** Classification of internal groove parameters based on the number of findings

Type	Number of internal groove findings	*n* (%)
Type 0	None	0 (0%)
Type I	One	11 (15.7)
Type II	Two*	18 (25.7)
Type III	Three*	30 (42.9)
Type IV	Four or more*	11 (15.7)

* Any combination

## Discussion

### Internal groove findings (OF, MF, GF)

During skull base surgery, and discussing pathologies involving deep intracranial structures such as the DVSs, it is essential to recognize and master the role of the emissary veins and the foramina through which they pass. Regarding the posterior cranial fossa, the major emissary veins are the mastoid, occipital, and condylar, which pass through the MF, OF, and posterior condylar foramen, respectively.

### Occipital foramen

Alghamdi et al. [10] reported that the prevalence of OF was 92/95 or 96.3%, mostly bilateral (*n* = 75; 78.9%), followed by unilateral OF (*n* = 10; 10.52%). Murlimanju et al. [11]. obtained a lower prevalence, closer to the one reported herein. They identified an OF in 11/78 cases or 14.1%, this foramen being located on the left side (*n* = 5; 6.4%), right side (*n* = 4; 5.1%), or classified as a median foramen (*n* = 2; 2.6%), using the midline and external occipital crest as references [11]. However, those studies and others in the literature tend to report the location of the foramina externally rather than by looking at the posterior cranial fossa internally. The latter is vital for determining the exact location of the internal opening, which can vary depending on the interosseous course of the canal; or the foramen could be a non-communicating foramen that opens into the diploic space. In the present study, internal openings of OF were found on 20 sides of the TS groove, more frequently on the right TS groove (*n* = 17; 85%), while on 14 sides the OF was identified on the occipital bone, but as it was initially defined.

### Mastoid foramen

Several studies have been dedicated to this foramen and its intricacies [12–14]. It is variable in multiple characteristics including external opening location, diameter, patency, canal course, and number of accompanying foramina. Louis et al. [13] found it more frequently on the right side (*n* = 196; 98%) than the left (*n* = 144; 72%). Similarly, Pereira et al. [14] reported MF to be more common in male (*n* = 22 sides; right: 90%; left: 85%) than female (*n* = 40 sides; right: 72.7%; left: 81.8%) skulls. Chaiyamoon et al. [15] recently described three classifications: modified Louis anatomical and surgical classifications of the MF [13] based on location, prevalence, communication with the SS and the exact entrance into the SS groove; and another classification of the mastoid emissary vein (MEV) based on its intraosseous course. The most prevalent MF was Type I (*n* = 22; 50.0%), defined as one external opening: one connecting with the SS groove, Type Ia: (*n* = 20; 45.5%); located either on the occipitomastoid suture, Type Ia-1: (*n* = 19; 43.2%), or parietomastoid suture, Type Ia-2: (*n* = 1; 2.27%); and one non-connecting MF, Type Ib: (*n* = 2; 4.5%). The course of the MEV was classified into three types, ascending, horizontal, and descending in a sagittal plane (coupled with posterior-to-anterior in an axial plane), the most common among which was horizontal (*n* = 12; 57.1%), followed by descending (*n* = 7; 33.3%).^15^ Zhou et al. [16] classified the course as straight (*n* = 23; 57.9%) or curved (*n* = 15; 42.1%). Importantly, independently of its course, both studies found that the internal opening of the MF was always located on the SS groove, as also described herein.

### Granular foveolae

These are defined as bony depressions created by arachnoid granulations and have been mostly described in the superior sagittal sinus, followed by the TS and SS. It is important to know that these bony depressions could also be associated with venous sinus diverticula, which are mostly identified on imaging of the sigmoid sinus. They tend to differ in their projections; however, detailed differentiation between these two causes of bony depressions has yet to be determined. Furthermore, recognition of arachnoid granulations is useful in order to differentiate them from pathologies such as thrombosis. It has been proposed that these structures determine the reabsorption of cerebrospinal fluid (CSF) [17, 18]. Gururi et al. [17] found in 110 skulls (220 sides) that the prevalence of GF in the SS groove was 3.6% (left: 0.9%; right: 2.7%). Interestingly, they reported no cases of bilateral GF. Moreover, Tsutsumi et al. [18] using contrast MRI reported arachnoid granulations located at the TS (right: *n* = 41 or 40.2%; left: *n* = 38 or 37.3%), SS (right: *n* = 18 or 17.6%; left: *n* = 19 or 18.6%), straight sinus (*n* = 36; 35.3%), and confluence of the sinuses (*n* = 21; 20.6%). Other studies have used non-contrast MRI.

### Surgical and clinical considerations

#### Internal groove findings (MF, OF, GF = arachnoid granulations)

The high variability of the different characteristics discussed in this article is important not only for endovascular and open procedures but also for better understanding of the configuration of vessels at the posterior cranial fossa and of how the intracranial venous system could adapt in pathological scenarios. For instance, the MF/MEV has been used in endovascular transvenous coil embolization in patients with vein of Galen malformations, and transcranial puncture has been deployed to treat dural arteriovenous fistulas and during skull base procedures including mastoidectomy and epitympanectomy, and in retro-sigmoid, transcondylar, and suboccipital approaches [16, 19, 20]. Furthermore, it is well known that emissary veins affect CSF hemodynamics because when they are present they can provide complementary venous outflow from the intracranium. Therefore, they could help significantly in regulating blood pressure and protecting the brain when intracranial pressure is raised, as in cases of CVST, VSS, and IIH [12–16]. However, the combined and complex role of the simultaneous presence or absence of emissary veins, arachnoid granulations, anatomical variations, etc. in either healthy subjects or pathological cases is not well understood. The aim of this study was to provide data from the skulls to elucidate this role. All of the features evaluated were found bilaterally in many specimens, excluding the MF. The classification based on the number of internal groove findings helps to explain that three findings (type III) were mostly restricted to the posterior cranial fossa with different combination patterns. The documented preponderance of internal groove findings leads us to hypothesize that they could be complementary in regulating and adapting the intracranial venous system efficiently. In addition, the lack of complementary function when MEV, occipital emissary vein (OEV), GF, TS, etc. are absent in combinations yet to be determined could predispose to the development of CVST or IIH. Furthermore, as discussed, emissary veins and arachnoid granulations are usually associated with benefits for the intracranial venous system, though certain situations have been described in which complications predominate and outweigh the benefits. When a MEV is sufficiently dilated (> 4 mm identified during preoperative CT), potentially because there is no complementary adaption from the intracranial venous system (absence or malfunction of emissary veins or arachnoid granulations), it could easily be injured at either its external or its internal opening, where the SS could also be torn. Dilated MEVs have also been implicated in pulsatile tinnitus cases where treatment could be warranted, in intracranial infections as direct pathways for bacterial seeding, in air embolism or thromboembolism when bone wax migrates to the SS, etc [21–23]. There are also cases associated with the other internal groove findings that support the lack of complementary venous adaptation, such as large OEVs misdiagnosed as arteriovenous fistulas, and large arachnoid granulations associated with VSS and IIH [24, 25]. 

### Absent TS groove and bony prominence

The TSs are still small around the 11th and 12th weeks of embryo development, until they undergo probably the most critical stage of their embryogenesis: the enlargement or ballooning phase. During this stage, from the 17th until 28th weeks, they progressively enlarge but can increase or decrease in size variably. These changes could potentially generate abnormalities such as septum formations, irregular margins and diameters, and even partial or complete absence of segments [26–28]. Correspondingly, structural developmental abnormalities including dural sinus septum, hypoplastic-aplastic-absent TS, TS stenosis, etc. have been associated with pathologies such as CVST of the TS, pulsatile tinnitus, VSS, and IIH [25, 29–31]. Arauz et al. [32] investigated the association between TS hypoplasia and CVST in 20 adults with isolated TS thrombosis and 43 controls using neuroimaging. They found a statistically significant association between hypoplasia and ipsilateral TS thrombosis (right TS hypoplasia, *p* = 0.002; left TS hypoplasia, *p* = 0.008), with a RR of 3.8 (95% CI 1.3–10) for right hypoplasia and 7.5 (95% CI 1.1–48) for left hypoplasia. Similarly, Connor et al. [33]. used neuroimaging to study TS stenosis in patients with (*n* = 14) and without (*n* = 19) IIH, looking at the relationship of TS stenosis to bony groove dimensions and elucidating the etiology of IIH. They showed that the height (Height_bone_ /height_sinus_) of tapered TS stenoses seen in subjects without IIH and most patients with IIH was associated with proportionately small or absent bony grooves, suggesting that the etiology of TS stenosis could involve primary or fixed (intrinsic) causes. In contrast, other group of patients with IIH demonstrated disproportionately large bony grooves, suggesting a secondary or acquired (extrinsic) narrowing. Interestingly, in four cases with height ratio > 1.5 (*n* = 8), discordance was identified. The authors proposed that this outcome could be explained by the presence of associated draining cortical veins or arachnoid granulations [33]. In this anatomical study we documented an absent TS groove with a prevalence of 29.85% (notably, this finding was usually seen in different combinations, but was the most common among isolated internal groove findings). In addition, a novel finding was identified and defined as a BP, potentially the product of an anomalous membranous or endochondral ossification, though its cause has yet to be investigated [34]. To our knowledge, this is the first study to describe the BP for the TS groove with a prevalence of 15.7%, located on the left side in almost all cases (*n* = 10; 90.9%). The BP effaced the groove for the TS, suggesting the possibility of an absent TS in the affected segment. However, we also thought that subjects with a TS who also had this variation could develop extrinsic compression of the TS by the underlying prominent bone, potentially leading to VSS, alterations in venous flow, and associated pathologies.

### Limitations

This study was performed using skulls, so owing to the lack of direct comparison with soft tissue it precludes exact determination of the documented findings. However it provides a basis for future studies investigating this relationship. The lack of clinical history and sociodemographic data from the specimens constitutes another limitation, since we considered and discussed the findings as normal, without considering potential comorbidities or previously diagnosed pathologies that could have affected the skulls.

## Conclusions

DVS pathologies most commonly involve the TS and their etiology is contentious. The multiple internal groove findings discussed in this study, along with their intricacies, provide a better understanding of the complex and integral role that these features could potentially have when they are present or absent simultaneously in normal patients and in those with pathologies such as VSS, IIH, pulsatile tinnitus, and CVST. To our knowledge, a BP that effaces the TS groove has not previously been reported; it could possibly be an extrinsic cause of TS compression.

## Data Availability

No datasets were generated or analysed during the current study.
